# Diseases of the Antrum of Highmore

**Published:** 1900-05-15

**Authors:** C. H. Oakman

**Affiliations:** Detroit, Mich.


### DISEASES OF THE ANTRUM OF HIGHMORE.

BY C. H. OAKMAN, D.D.S., DETROIT, MICH.

It is my purpose in this paper to relate a few cases of the above which have come under my observation.

The first, a male of forty-three, came to me through his physician. He was in a very weakened and anæmic condition, complained of a heaviness in the right side of his head, and a desire to rest his head on his hands; the right cheek was considerably swollen and a distention of the outer plate of the superior maxillary; the first bicuspid and first and second molars badly decayed and suppurating at the necks of the teeth; there was practically no circulation existing. The soft tissue was of a gangrenous and sluggish nature.

Between the second bicuspid and third molar a lancet could be inserted without pain to the patient. From the appearance I was satisfied that the antrum was involved. The history of the case extended over a period of twelve years

At no time previous to my operating on his jaw did he ever complain of severe pain in the region of the antrum.

Before he came to me he had two intestinal hemorrhages at periods of about two years apart. The whole alimentary tract seemed to be affected. He had both stomach and intestinal indigestion. His physician was of the opinion that there was some abnormal growth in the intestines

After one year's treatment the second attack of hemorrhage came on, due no doubt to weakening of the parts caused by pus absorption.

When I took charge I immediately extracted the teeth (or rather roots). This was followed by a copious flow of blackish, odoriferous pus. Inserting a probe into the lingual sockets of the molars and found I could enter the antrum without any resistance.

On further exploration I found that the floor of the antrum was necrosed. Owing to the condition of the patient I could not prolong the operation further, as it was not safe to administer a profound anesthetic, so I irrigated with warm boracic acid solution and dismissed him until the next day.

The second day found the patient somewhat worse, undoubtedly due to pain and anxiety. This day gave another treatment of boracic acid water and continued same for about ten days, and then I was able to remove considerable diseased bone.

Seeing the patient daily and keeping the parts thoroughly irrigated for several weeks, I was able to remove from time to time small pieces of bone. Then using the large antril drills I made a liberal opening removing a greater portion of the floor of the antrum, using diluted peroxide and 2 percent carbolic acid, tamponing the antrum with boracic acid gauze.

I have found a hard rubber plug fastened to the adjacent teeth can be held firmly in place and removed by the patient at any time, cleansed and replaced.

During the treatment of this case I felt called upon to use heroic measures and resorted occasionally to the use of bichloride of mercury, 1-5000, but after every treatment with it I found it so irritant as to cause the patient considerable pain and discomfort, also causing an excessive flow of pus. On two occasions he had to return to the office after having reached home.

Treating the antrum with 2 percent solution of carbolic acid, the patient's sufferings were relieved in a few moments, since which time I have discarded irritating substances altogether.

From the middle meatus of the nares, the natural opening of the antrum being quite large, I could irrigate very thoroughly and I seldom found it clogged.

The patient was under constitutional treatment for several months. Hypophosphites, iron and other tonics were given with apparent good results.

Finally, upon exploring in the region of the third molar (buccal surface), I found a fistulous opening. The process surrounding it was diseased, so I decided to extract the third molar. This left no teeth posteriorly to the cuspid. This was about the fifth month of treatment. At that period the patient began to show marked signs of improvement. Suppuration would cease for a week at a time, then an exudation more marked than before would appear.

I much prefer packing the antrum with gauze for if there is any pus present the gauze will absorb it, and when removed its presence can readily be detected. Not so in irrigating through a tube, as small quantities may, during irrigation, pass through the tube unnoticed.

On the second anniversary of the patient's first visit to my office I discharged him as cured.

He had gained in flesh and his health was better than any time within twelve years. When the patient first came to me he was just able to walk. The contrast was very marked when I discharged him cured.

*Case* 2.—The second case was that of a young woman, twenty-eight years of age, who complained of pain in the region of the second upper left molar. The tooth was sensitive to touch, apparently a case of pericementitis; it contained a large amalgam filling.

Upon inquiry, the patient stated that the pulp had been removed and canals filled. I removed the filling, treated the canals and applied a counter-irritant to the gum. The condition remained unchanged and I was unable to give her any permanent relief.

Further inquiry revealed the fact that the patient had had the left antrum operated upon six years previous. I extracted the molar and inserted a fine probe into the lingual canal and thin pus flowed out. I then concluded that the antrum was the cause of the difficulty.

The first molar being absent, I was enabled to make an opening half an inch or more in diameter. Irrigated with warm sterile water and packed the antrum with boracic acid gauze.

The patient also complained of a very bad odor after blowing her nose. The left eye was congested and the vision considerably impaired. Before coming to me she had treated with her oculist a year, using several lenses, none of which seemed to aid her for any length of time.

During the first two months' treatment with me she was able, without the use of glasses, to follow her vocation, of which needle work in colors comprised the greater part. For weeks the discharge of pus seemed to increase at each treatment, then it would subside for several days and start again to flow as freely as before.

About the fourth month she complained of an inflamed condition of the right eye. The lachrymal gland was also sensitive to pressure and continuing along the lower lid toward the malar bone; it appeared to me that the right antrum was infected by the pus from the opposite side.

After careful observation, I concluded to open into the right antrum. Upon penetrating the floor with a large antral drill, considerable pus flowed out. I continued to treat with boracic acid, dilute peroxide of hydrogen, nitrate of silver, 2 percent once a week, and carbolic acid, 2 percent, daily.

About the fifth month the patient was enabled to do some close work without her glasses. The several lenses were of no use to her. Having thorough drainage from both antra the oculist was enabled to fit her eyes perfectly for new glasses, the source of the trouble having baffled him for several months.

This being such an obstinate case, when seven months elapsed I was compelled to enlarge the orifice of the canals and curette both antra, treating as before mentioned, and insufflating with aristol, boric acid and packing with boric acid gauze. I continued this treatment for five months longer. Between the tenth and twelfth month the discharge ceased for two weeks.

About that time I was congratulating myself that the patient was nearly cured when it broke out anew, leaving me at my wit's end as to what further treatment would be advisable. After consulting authorities on oral surgery, I found I could do little more than I had already done.

At the end of a year's treatment the patient was taken ill with la grippe, became low-spirited and melancholy in spite of various tonics and words of encouragement. It is a wonder to me how patients so afflicted have as much courage as they do. The frequent dropping of pus through the posterior nares, the discharge through the nose is to them a constant reminder of their affliction, it being necessary to carry with them antiseptics to disguise a fetid breath.

It should be constantly borne in mind that in cases of this kind the bowels should never be neglected. Saline cathartics and other laxatives should be prescribed, especially in warm weather, thus keeping the stomach and intestines from being disordered, for the quantity of pus taken into the stomach during the night will naturally nauseate and disturb the patient.

Two years have elapsed and the patient is not cured. It is my opinion that in this case the frontal sinus is affected.

It has been recently demonstrated beyond doubt that there is a connection between the antrum and frontal sinus, the infundibulum.

As to general health the patient is much improved.

*Case* 3.—The next was a young woman twenty-four years of age, who came to me with an abscess on the right anterior portion of the palate extending to the medium line. It was larger than a hickory nut, probably not quite as deep, but covering a greater area.

The patient could give no definite history of the case further than having fallen upon the ice ten years previous, and injuring her upper incisors. This had loosened the teeth but they soon became firmly attached. She had never experienced any further discomforture The pulp had died in the right incisor and to this tooth is due the origin of the disease.

Upon questioning the patient, she said she had noticed a yellowish discharge from the right nostril but thought it a form of catarrh, and received treatment from her physician which gave but temporary reliet.

After lancing the abscess and washing out with warm sterile water, I inserted a probe and found the palatal bone badly necrossed, and a cleft about a quarter of an inch along the medium line posterior to the incision I had made.

I dismissed the patient until the next day. When I drilled through the lingual surface of the incisor considerable pus made its escape. Inserting a broach into the root canal I could find no resistance beyond the apex of the tooth, so I decided to amputate the root after which I found the condition much worse than I had expected.

It was my desire to save the teeth if possible, but finding my efforts of no avail, I extracted the right central, removing the diseased bone from around the left central and found it also had to be extracted. After a few moments use of the probe, I saw that it would be necessary to remove considerable portion of the right palatal bone.

In a few days the patient was anesthetized and a portion of the bone to the extent of about an inch and a half removed with a curette and a large antral drill.

The next day I called at the patient's home, found her with fever and very weak. After removing the gauze, the stench was so marked that the windows had to be lowered. She complained of much pain in her head and face. I made visits to her home daily for a week, and at the end of which time she was able to come to the office.

As in other cases, the patient's system was weakened from constant pus absorption. She did not seem to recover from the shock of the operation.

Constitutional treatment was advised, (Fellow's syrup of hypophosphites and extract of malt gave her the most strength.)

Considering the cleft in the palate and the patient's condition, I thought it might be tubercular. Had the pus examined, but fortunately nothing of this nature was found. Continued to treat the patient for more than a year. At that time she was about to make a visit to Chicago. Taking advantage of this opportunity, I recommended the patient to Dr. T. W. Brophy, the noted oral surgeon, in the meantime furnishing him with a history of the case and asking his advice in the matter. In the course of our correspondence he informed me that he had made a thorough examination of the case, and advised a continuation of the treatment I had already given. As there was thorough drainage from the antrum and the palate, it was now only a question of time before the patient would recover.

Several weeks have elapsed and there is scarcely a sign of pus, and the orifice of both cavities are nearly healed.

*Case* 4.—The next was a male of thirty-two years of age, for whom I had done considerable operating and was about to prepare a bridge with first bicuspid and second molar for abutment on the upper left side. Found it necessary to extract the roots of the first molar. While waiting for the parts to absorb I noticed, at different times, pus dripping from the sockets of the buccal canals. With some difficulty I was able to insert a probe in the antrum. The pus came more freely after removing the probe.

Three days later I administered chloroform and gained a liberal opening, irrigating with warm sterile water and saturated solution of boracic acid and packed the orifice of the canal for the first week with boracic acid gauze.

The patient had received treatment for nasal catarrh from his physician during a period of ten years, which gave him only temporary relief.

He informed me that he had noticed considerable yellowish discharge from his right nostril, and occasionally a slight discharge from the left.

It seemed to me if the left antrum was affected, it should have been the reverse. After treating the case two months I concluded the right antrum was the primary cause of the trouble.

After carefully questioning the patient he remembered having a slight sensation of pain extending from the nose along the lower right eyelid and a radiating pain in the region of the frontal bone; these sensations he had noticed for years, but never had the pain become distressing.

That heavy and uncertain feeling existing in that region was always relieved after a discharge from the right nostril, the heaviness due no doubt to the antrum being filled with pus and the distention of gases.

A second time administered chloroform, the first and second molars being absent on the upper right side gained entrance through the floor of the antrum at the most dependent point, which is usually the second molar. Made an opening a little over half an inch, drilling on a line with the inner right canthus The pus was dark and odoriferous. Curetted the antrum and removed a thick pus accumulation, washing the parts with warm sterile water and diluted peroxide of hydrogen, insufflated with aristol and boracic acid and packed tightly with gauze.

During the next three months continued the same treatment, with the addition of glyco-thymoline and protargal, the latter being highly recommended in a recent article in the *Digest*, which advocated its great efficiency in antral diseases.

Notwithstanding all that was said of its non-irritating properties, I have practically demonstrated it to be an irritant, except in 1 or 2 percent solution. Thymoline is less irritating in 25 percent solution.

After five months' treatment there was practically an absence of pus for a month, due no doubt to my strong adherence to non-irritating substances, of which there is nothing better than a saturated solution of boracic acid. After one treatment of protargal, 10 percent solution, there was more pus visible than at any time within a month. If it were not an irritant, it would not have aggravated the mucous membrane.

( *To be Continued.* )

---

PYROZONE AS A STYPTIC COAGULANT.—Dr. H. J. Kauffer, in the April *Cosmos*, page 293, relates two cases of persistent hemorrhage which he controlled by applications of 25 percent solutions of pyrozone. He cleans the wound first and then applies the pyrozone on a plug of cotton. Pyrozone is rather an irritating caustic, and should be used cautiously.

